# Investigating the Fractal Dimension of the Foveal Microvasculature in Relation to the Morphology of the Foveal Avascular Zone and to the Macular Circulation in Patients With Type 2 Diabetes Mellitus

**DOI:** 10.3389/fphys.2018.01233

**Published:** 2018-09-05

**Authors:** Maja Kostic, Nathan M. Bates, Nebojsa T. Milosevic, Jing Tian, William E. Smiddy, Wen-Hsiang Lee, Gabor M. Somfai, William J. Feuer, Joyce C. Shiffman, Ajay E. Kuriyan, Ninel Z. Gregori, Sandra Pineda, Delia Cabrera DeBuc

**Affiliations:** ^1^Miller School of Medicine, Bascom Palmer Eye Institute, University of Miami, Miami, FL, United States; ^2^Department of Biophysics, University of Belgrade, Belgrade, Serbia; ^3^Retinology Unit, Pallas Kliniken, Olten, Switzerland; ^4^Department of Ophthalmology, Semmelweis University, Budapest, Hungary

**Keywords:** diabetic retinopathy, fractal analysis, foveal avascular zone, blood flow rate, fractal dimension

## Abstract

In this study, we examined the relationship between the fractal dimension (FD), the morphology of the foveal avascular zone (FAZ) and the macular circulation in healthy controls and patients with type 2 diabetes mellitus (T2DM) with and with no diabetic retinopathy (DR). Cross-sectional data of 47 subjects were analyzed from a 5-year longitudinal study using a multimodal optical imaging approach. Healthy eyes from nondiabetic volunteers (*n* = 12) were selected as controls. Eyes from patients with T2DM were selected and divided into two groups: diabetic subjects with mild DR (MDR group, *n* = 15) and subjects with DM but without DR (DM group, *n* = 20). Our results demonstrated a higher FD in the healthy group (mean, 1.42 ± 0.03) than in the DM and MDR groups (1.39 ± 0.02 and 1.35 ± 0.03, respectively). Also, a bigger perimeter, area, and roundness of the FAZ were found in MDR eyes. A significant difference in area and perimeter (*p* ≤ 0.005) was observed for the MDR group supporting the enlargement of the FAZ due to diabetic complications in the eye. A moderate positive correlation (*p* = 0.014, *R*^2^ = 43.8%) between the FD and blood flow rate (BFR) was only found in the healthy control group. The BFR calculations revealed the lowest values in the MDR group (0.98 ± 0.27 μl/s vs. 1.36 ± 0.86 μl/s and 1.36 ± 0.57 μl/sec in the MDR, DM, and healthy groups, respectively, *p* = 0.2). Our study suggests that the FD of the foveal vessel arborization could provide useful information to identify early morphological changes in the retina of patients with T2DM. Our results also indicate that the enlargement and asymmetry of the FAZ might be related to a lower BFR because of the DR onset and progression. Interestingly, due to the lack of FAZ symmetry observed in the DM and MDR eyes, it appears that the distribution of flow within the retinal vessels loses complexity as the vascular structures distributing the flow are not well described by fractal branching. Further research could determine how our approach may be used to aid the diagnosis of retinal neurodegeneration and vascular impairment at the early stage of DR.

## Introduction

Diabetes mellitus is one of the leading causes of vision loss (Diabetes Fact Sheet, 2017). Central vision loss, which is the most common impairment related to diabetic macular edema (DME), has a big impact on quality of life (Bourne et al., 2013; Korobelnik et al., 2014). The worldwide prevalence of DM is predicted to grow to 430 million patients by 2030, according to the World Health Organization (Korobelnik et al., 2014). Diabetes prevalence has also increased at a faster rate in lower income countries when compared to wealthier nations countries (Diabetes Fact Sheet, 2017). Also, rates of retinopathy are higher among people with type 1 diabetes mellitus (T1DM), individuals with longer duration of diabetes, and Caucasian populations (Yau et al., 2012; Bourne et al., 2013; Korobelnik et al., 2014). There is also a correlation between lower socioeconomic status and higher rates of retinopathy (Yau et al., 2012; Bourne et al., 2013).

Microaneurysms, capillary nonperfusion, and ischemia within the retina are the characteristic pathological features of DR (Elman et al., 2010; Hwang et al., 2015, 2016; Ishibazawa et al., 2015). These pathophysiologic changes can be associated with several complications, such as DME and diabetic macular ischemia (Beltramo and Porta, 2013; American Academy of Ophthalmology., 2014; Varma et al., 2014; Couturier et al., 2015; Das et al., 2015; Bradley et al., 2016). These complications compound impaired blood and oxygen supply of the neuroglial tissues of the retina. The expression of vascular endothelial growth factor (VEGF), which acts as an angiogenic agent and increases vascular permeability, is enhanced in the hypoxic environment (Choi et al., 2013). Diabetic maculopathy is caused by a combination of both VEGF-mediated factors and mediators (Agemy et al., 2015; Mastropasqua et al., 2015; Gorczynska et al., 2016). High glucose levels lead to microvasculopathy with alterations in the blood-retinal barrier, causing pericyte loss and endothelial cell-cell junction breakdown (Kaur et al., 2008). This capillary disruption increases vascular permeability and the pooling of fluid within the plexiform layers of the retina and the subretinal space (Ishibazawa et al., 2015). These pathophysiological changes can result in a gradual loss of visual acuity.

The FAZ is the macular region that is most susceptible to retinal changes in individuals with diabetes (Choi et al., 2013; Varma et al., 2014). The FAZ is the central part of the macula and is surrounded by interconnected capillary beds. This vascular network terminates in the central macula forming a vascular ring-shaped border with an average diameter of 500–600 μm (de Carlo et al., 2015a,c). In some patients with DME, central visual loss may be due to edema as well as ischemia occurring due to capillary dropout sufficient to increase the FAZ area (Choi et al., 2013). Most DR patients will not experience vision changes until late-stage disease, therefore, early detection and immediate intervention may better preserve vision (Aiello, 2003). Consequently, early detection and accurate staging are critical for determining optimal management. DME treatments have been developed, including focal or grid photocoagulation and anti-VEGF therapy, both of which have been demonstrated to be effective (American Academy of Ophthalmology., 2014; Couturier et al., 2015; Wiley et al., 2016). Anti-VEGF therapy has also been shown recently to slow the progression and, in some cases, reverse the degree of ophthalmoscopically observed nonproliferative retinopathy (Ip et al., 2012, 2015). However, the pathology involved in the retinal hemodynamics throughout the course of DR is not completely understood (Pemp and Schmetterer, 2008; Kur et al., 2012; Stitt et al., 2016).

Various diagnostic techniques exist to assess the structure and function of the retina. Ophthalmoscopy and fundus photographs are standard techniques used in DR, with the fundus photographs being comparable to ophthalmoscopy under dilated pupils while remaining cost effective for screening in diabetes clinics (Lee et al., 1993). Fluoroscein angiography is also recognized as a useful tool for healthcare professionals diagnosing and treating DR (Agemy et al., 2015). However, it requires venipuncture which may lead to allergic reactions and, in rare cases, death due to anaphylaxis (Yannuzzi et al., 1986). In addition, the technique is costly and time-consuming, requiring up to 30 min only for image acquisition itself (Matsunaga et al., 2014; Sim et al., 2014; Di et al., 2016). Nevertheless, it has historically been the standard in the assessment of DR and DME. Retinal hemodynamic abnormalities and retinal oxygen metabolism have also been investigated in patients with DR (Kohner et al., 1995; Bursell et al., 1996; Calles-Escandon and Cipolla, 2001; Schram et al., 2005). For example, the retinal function imager (RFI) (Optical Imaging Ltd, Rehovot, Israel) is a noninvasive imaging technique that has been used to investigate the microcirculation in the retina of patients with DM (Grinvald et al., 2004; Nelson et al., 2005).

Research in different areas of complications associated to DM is constantly evolving (Campagnoli et al., 2017; Somfai et al., 2018). The search for risk biomarkers characterizing preclinical abnormalities is fundamental to fast-track the discovery of novel treatments. For example, FD is one of the vascular architectural parameters commonly used to quantify changes in the retinal branching pattern and vascular density due to disease progression (Lim et al., 2009; Cosatto et al., 2010). Fractal geometry studies of the retinal vasculature can be performed by fractal analysis, a mathematical method used to measure complexity in natural phenomenon (Mandelbrot, 1982). The concept of fractal geometry was first described by Mandelbrot in 1989 (Smith et al., 1996; Fernández and Jelinek, 2001; Di Ieva et al., 2015). Later, Family et al. introduced this method in ophthalmology and since then, interest in studying the association between the FD of the retinal vasculature and disease severity and progression has dramatically increased (Family et al., 1989; Fractals medicine., 1991; Cheung et al., 2009; Grauslund et al., 2010; Yau et al., 2010; Aliahmad et al., 2014; Broe et al., 2014). Although, the retinal vasculature tree could be quantified with various methods of fractal analysis (Stosic and Stosic, 2006; Macgillivray et al., 2007), the digital retinal images could be investigated through complexity, space-filling, shape, and tortuosity of retinal blood vessels. These characteristics could be quantified by the box-counting method of fractal analysis (Milošević, 2015).

In this study, we examined the relationship between the fractal dimension (FD), the morphology of the foveal avascular zone (FAZ) and the macular circulation in healthy controls and patients with type 2 diabetes mellitus (T2DM) with and with no diabetic retinopathy (DR). Our results suggest that the FD of the foveal vessel arborization could provide useful information to identify early morphological changes in the retina of patients with T2DM.

## Methods

### Study population

The study was approved by the Institutional Review Board (University of Miami, Miami, FL, USA). The research adhered to the tenets outlined in the Declaration of Helsinki and written informed consent was obtained from each study subject. In this prospective study, enrollment was offered to patients with DM referred to the comprehensive ophthalmology clinic that had DR up to early treatment diabetic retinopathy study (ETDRS) level 35 and without macular edema, as well as diabetic patients with no retinopathy and healthy individuals (Group ETDRSR, 1981).

Patients with proliferative disease, clinically significant macular edema (CSME), and anatomic abnormalities that might confound the evaluation of macular architecture (such as glaucoma, vitreoretinal traction, and epiretinal membranes) were excluded. Patients with medical conditions that might affect visual function, those taking medications that might affect retinal thickness (e.g., chloroquine or anti-cholesterol agents containing niacin), recent cataract surgery, previous vitrectomy, or unstable blood sugars were also excluded.

The routine ophthalmic examination was carried out with dilated fundoscopy and patients were divided into two groups based on the absence of DR (DM group) and presence of mild DR (MDR group). Any eyes with more severe DR (i.e., greater than ETDRS level 35) were excluded from the study. Study subjects (age-matched) were selected from a 5-year longitudinal study based on the quality of the overall imaging data required to perform all analyses. Table 1 shows the demographics of the study population. A total of 47 study participants (58 eyes) were identified with good quality images from the RFI system.

**Table 1:** Study Participant Demographics.

**Descriptor**	**Healthy**	**DM**	**MDR**
Number of patients (Male/Female)	12 (3/9)	20 (6/14)	15 (8/7)
Number of Eyes (OD/OS)	13 (5/8)	28 (10/18)	17 (9/8)
Mean age ± SD, years	54.08 ± 7.71	52.64 ± 7.79	52.63 ± 5.79

### Hemodynamic analysis

This analysis was performed by the RFI system, which is based on a standard fundus camera extended by a customized stroboscopic flash lamp system. A green (“red-free”) light with a spectrum of 548 ± 75 nm is used for illumination and the interval between consecutive flashes is typically 17.5 ms. One session of RFI data consists of 8 images with a resolution of 1,024 × 1,024 pixels in an area of 4.3 × 4.3 mm or 7.2 × 7.2 depending on the choice of field of view (20° or 35°) during the imaging acquisition. In this study, all the images were captured with the setting of 20° field of view (FOV) at a resolution of 4.3 microns/pixel. The heartbeats of the patient were monitored with a finger probe sensor and the image acquisition was synchronized with the cardiac cycle to neutralize the effects of pulsation on arterial blood flow velocity (BFV) (Grinvald et al., 2004; Nelson et al., 2005; Tian et al., 2016).

Once the image was acquired, the RFI built-in software generated (a) the flow movie (a.k.a. “ratio video”) through differential processing so that the motion of individual clusters of red blood cells can be followed by the human eye; and (b) a non-invasive capillary perfusion map (nCPM) was generated through analyzing the difference of pixel intensities in adjacent frames (Nelson et al., 2005; Izhaky et al., 2009). A good quality scanning session is characterized by sharp vessel borders on the raw fundus images, clear red blood cell movement along the vessels on ratio videos and a visible capillary network on the nCPM. Therefore, images were evaluated for optical quality, exposure and focus. We obtained 3 or more good-quality sessions for each eye by the same experienced photographer with at least 5 good images per session that were selected for further analysis using a custom-built software (Tian et al., 2016). In our method, the BFV is calculated by maximizing cross-correlation of intensity profiles between adjacent frames and the BFR is computed by multiplying the BFV with the cross-sectional area (Tian et al., 2016). All BFR measurements were obtained for the overall arteries, overall veins and overall vessels (i.e., arteries and veins) for each study group.

### Fractal analysis

**Figure 1:**
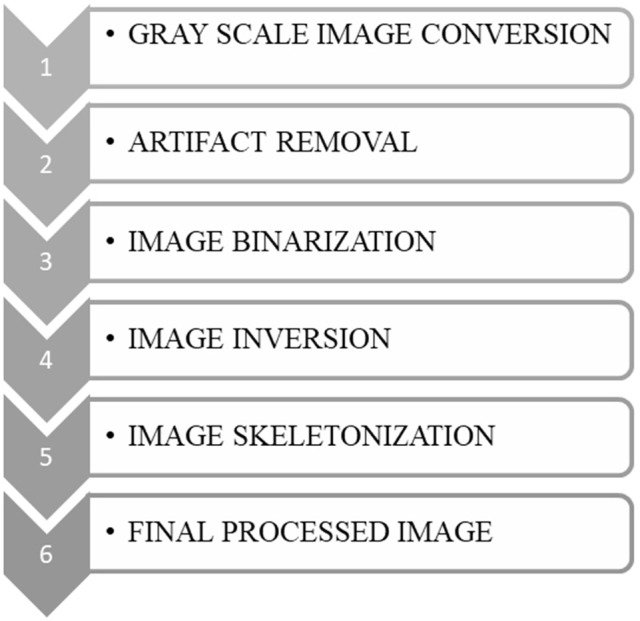
Sketch diagram of skeletonized box dimension (*D*_skel_) obtained by the box-counting method.

The fractal analysis of the retinal vascular network was performed using the box-counting method (Smith et al., 1996; Fernández and Jelinek, 2001; Milošević, 2015, 2016; Rajkovic et al., 2017). The RFI images were imported in Image J (National Institutes of Health, Bethesda, MD) and used to calculate the FD after grayscale format conversion (Figure 1). The box counting method generates data by “covering” the object with a rectangular coordinate grid and breaking the data into boxes and then analyzing the subsets by counting the number of boxes (Smith et al., 1996; Fernández and Jelinek, 2001). The lower and upper box-dimensions of a subset *F*⊂*R*^*n*^ are respectively defined by

(1) dimB(F)=limδ→0log Nδ(F)-log δ,dimB(F)=limδ→0log Nδ(F)-log δ

and if lower and upper values are equal, then the common value is referred to as the box-counting dimension of *F* and is denoted by

(2) dimB(F)=limδ→0log Nδ(F)-log δ

Where *N*_δ_(*F*) can be the smallest number of cubes of side δ (naturally, in 3D) that covers *F* or the largest number of disjoint cubes of side δ with centers in *F* (Falconer, 1989).

When plane-projection of the object is analyzed, this method measures FD by covering the image with a rectangular coordinate grid with cell size *r* and counts the number of boxes, where the cell size is expressed as the number of foreground pixels. The number of squares *N*(*r*) needed to cover the image is given by a power law

(3) $$\begin{array}{l} N\left(r\right)=const\cdot r^{-D} \end{array}$$

Where *D* is the box dimension, calculated as an absolute value of the slope of the log-log relationship between *N*(*r*) and *r* as previously described in detail (Milošević, 2015).

### Morphology analysis of the FAZ

The foveal morphology analysis was performed on the nCPM composite images, which were generated from the individual scans taken with the RFI (Tian et al., 2016). These capillary maps were then used to gather information regarding the FAZ, which can be identified in the image as the central area where no vasculature is present. An active contouring model was used to identify and outline the FAZ (Nikolay, 2016; Bates et al., 2018). The active contouring program is run by constant user supervision; therefore, each run was reviewed ensuring that it accurately represented the FAZ. If it appeared to be inaccurate, the simulation was rerun with different parameters, specifically with a different region of interest drawn to capture the area better. If it was still not representative of the actual FAZ, the image was excluded from the analysis due to poor quality. This process was done blindly, in that the group of the patient was unknown. Following identification of the FAZ using active contouring, the images were analyzed using ImageJ (National Institutes of Health, Bethesda, MD) (Smith et al., 1996; Schneider et al., 2012). This software allows for the easy acquisition of data regarding the region of interest, including area, circumference, perimeter, and the maximum/minimum Feret diameter. Area, perimeter, and roundness are parameters that we used for this study.

### Ethics approval

The Human Research Ethics Committee of the University of Miami, Miami, FL, USA approved all protocols and methods described in this study. The research adhered to the tenets outlined in the Declaration of Helsinki. Informed consent was obtained from all participants following a thorough explanation of all test procedures.

### Statistical analysis

Linear regression was used to determine the relationship between the BFR, FD, and FAZ parameters among the three groups. A one-way ANOVA test was used to determine if there was a difference present in any group, and then a *post hoc* Kruskal-Wallis test was used to identify these individual group differences. In all cases, a *p*-value of 0.05 was used to define significance (Katz and McSweeney, 1980; Armstrong et al., 2000).

## Results

### Fractal dimension

**Figure 2:**
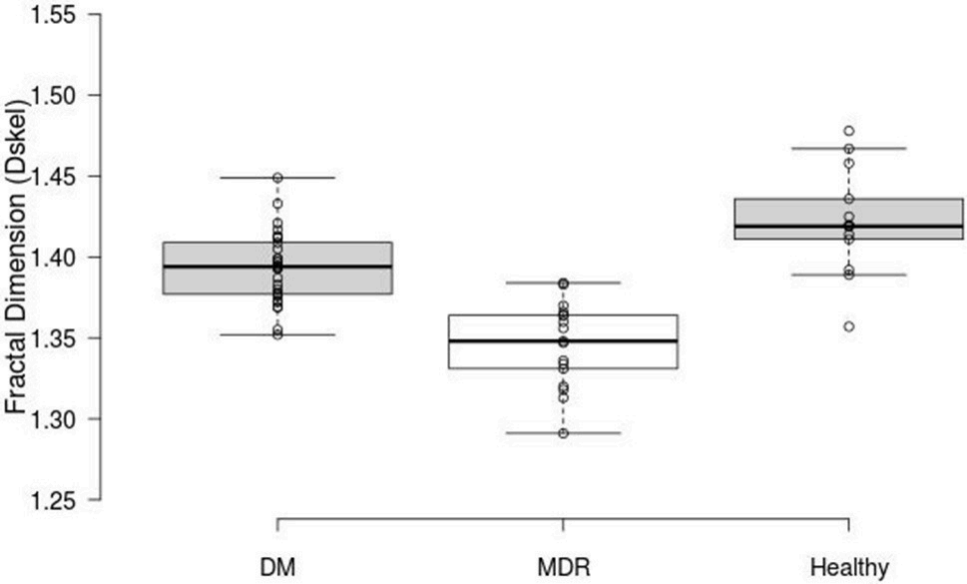
Box plots displaying the FD results. The middle 50% *(mean and 95% CI)* of the data groups are as follows: DM (1.3765–1.4105), MDR (1.3255–1.365), and Healthy (1.4015–1.447).

**Figure 3:**
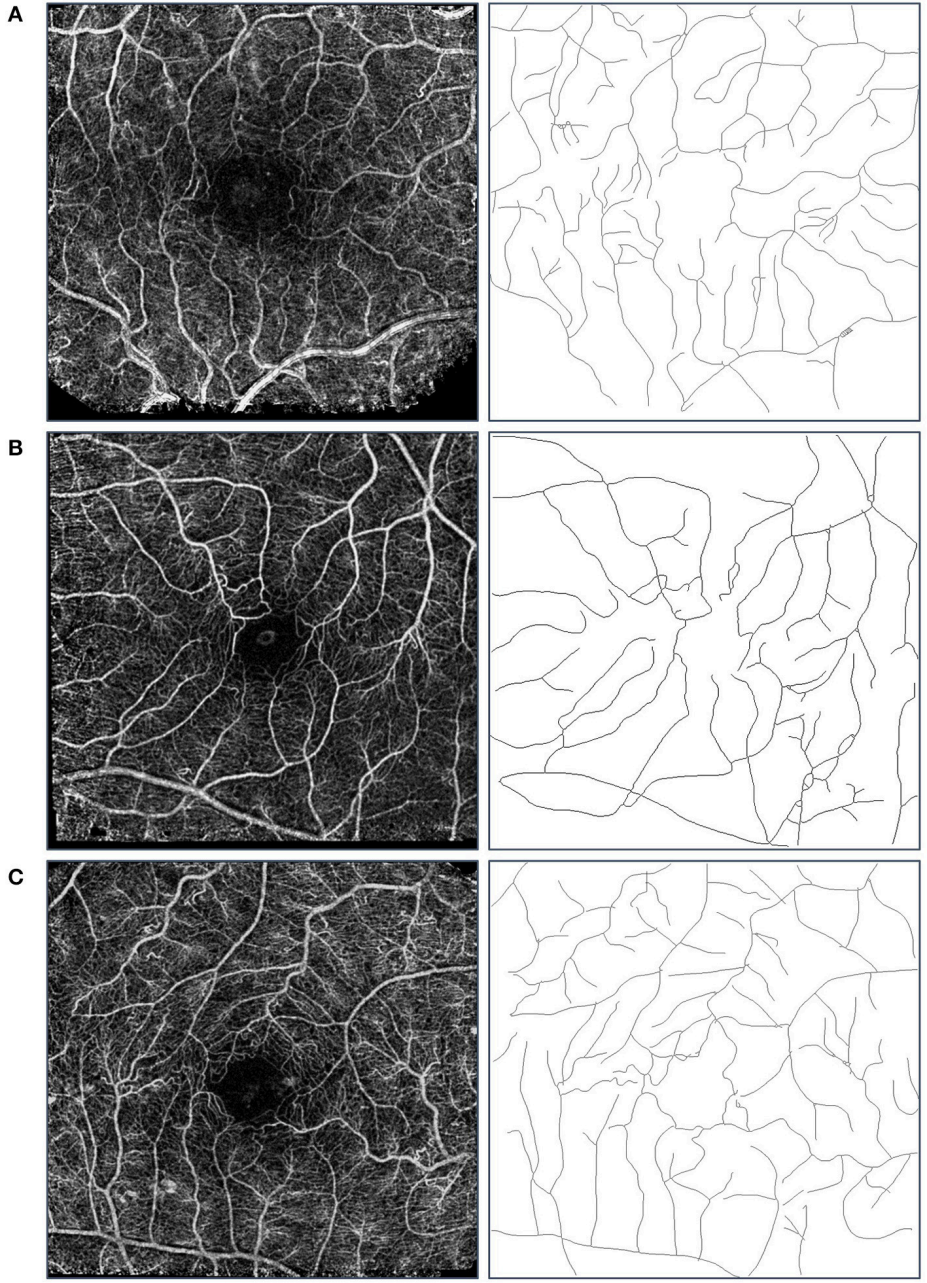
Sample images used in the fractal analysis. Images in the left column are the composite nCPM images generated from the RFI system, while those in the right are their respective skeleton images that were used in the fractal analysis. Row **(A)** is from the healthy group, Row **(B)** from the DM group, and Row **(C)** from the MDR group. We note that the blood vessel types used in the analysis were the major arterial and venous trees.

FD was calculated for the three study groups. ANOVA showed a statistically significant difference (*p* < 0.001) between all study groups. The highest FD values were obtained for the healthy group (1.42 ± 0.03) compared to those calculated for the DM and MDR groups (1.39 ± 0.02 and 1.35 ± 0.03, respectively) (see Figure 2). An example of the nCPM and corresponding skeleton images used in the fractal analysis is shown in Figure 3.

The intergroup differences measured with the Kruskal-Wallis analysis revealed a statistically significantly larger FD in the healthy group compared to the one obtained from the DM and MDR groups (*p* = 0.002). Also, a statistically significant difference (*p* < 0.001) was found between the healthy and the MDR group, as well as between the DM and MDR groups (*p* < 0.001).

### FAZ dimensions

The area, perimeter and roundness of the FAZ were calculated to characterize the morphology of the FAZ region (Bates et al., 2018) A summary of the results for the three study groups is presented in Table 2. ANOVA showed significance in all parameters with multiple significant differences in the pairwise comparisons as seen in Table 2.

**Table 2:** Results of the FAZ characterization for all three groups (first three columns show the morphological parameters).

**Descriptor**	**Healthy mean(±SD)**	**DM mean(±SD)**	**MDR mean(±SD)**	**Healthy vs. DM**	**Healthy vs. MDR**	**DM vs. MDR**
Area (mm2)	0.19 ± 0.05	0.21 ± 0.06	0.25 ± 0.06	0.26	<**0.01**	**0.02**
Perimeter (mm)	2.03 ± 0.24	2.16 ± 0.39	2.50 ± 0.41	0.29	<**0.01**	<**0.01**
Roundness	0.87 ± 0.07	0.84 ± 0.09	0.90 ± 0.06	0.21	0.31	**0.02**

*The p-values of the Kruskal-Walls test are shown in the last three columns) with significant results in bold. Note that roundness as defined by Image J – represents how closely a region conforms to a circle*.

### Blood flow rate

**Figure 4:**
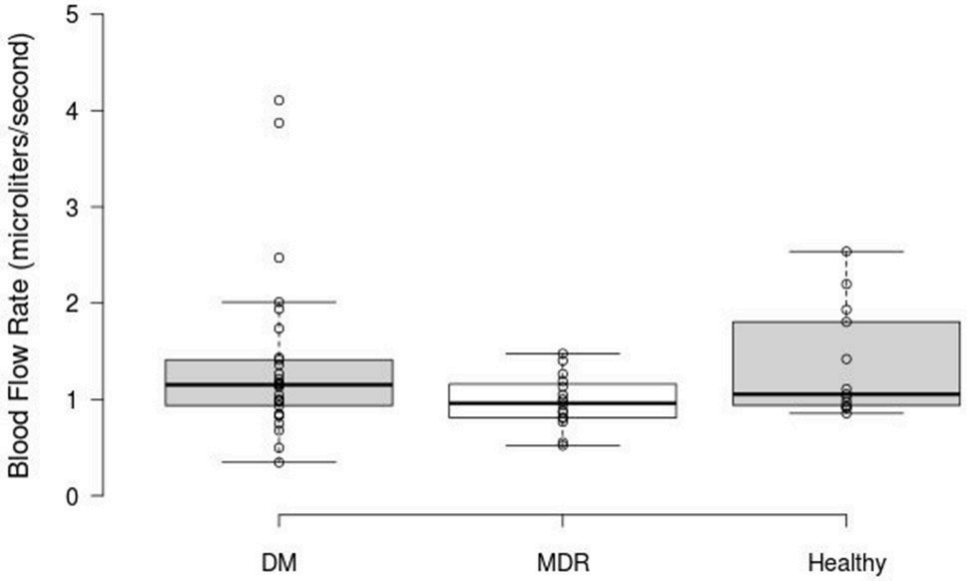
Box Plot displaying the BFR results obtained for the overall blood vessels in the three study groups. The middle 50% of the data groups are as follows: DM (0.89–1.42), MDR (0.809–1.17), and Healthy (0.85–1.868).

Figure 4 shows the BFR results obtained for the overall blood vessels (i.e., both arteries and veins) per study group. Our results showed 0.98 ± 0.27 μl/s, 1.36 ± 0.86 μl/s, and 1.36 ± 0.57 μl/s in the MDR, DM, and healthy groups, respectively. No significant difference (*p* = 0.2) was found in the ANOVA analysis.

### Relationship between the vascular FD, the morphology of the FAZ, and macular circulation

Linear regression results showed significant differences for the healthy group when analyzing the correlations between the vascular FD and BFR parameters (see Table 3). For all three groups of patients, FD was calculated and compared with BFR data for overall arteries, veins, and vessels. A summary of the results for all three study groups is presented in Table 3. Only the healthy control group showed a significant linear correlation between the FD and BFR parameters.

**Table 3:** Linear regression results obtained for all three groups after analyzing the correlations between the FD and BFR. Significant results (*p* < 0.05) are highlighted in bold.

**Group**	**Overall Arteries**	**Overall Veins**	**Overall Blood Vessels**						
	**Slope**	***R*^2^**	***p***	**Slope**	***R*^2^**	***p***	**Slope**	***R*^2^**	***p***
Healthy	0.04	0.45	**0.01**	0.03	0.31	**0.04**	0.04	0.44	**0.01**
DM	~0	< 0.01	0.8	~0	< 0.01	0.65	~0	< 0.01	0.75
MDR	0.01	0.02	0.58	0.03	0.14	0.16	0.03	0.10	0.24

*Results are presented as: Slope of linear regression, R^2^ value, and p-value. Slope listed as ~0 refers to a slope of less than 0.001*.

**Figure 5:**
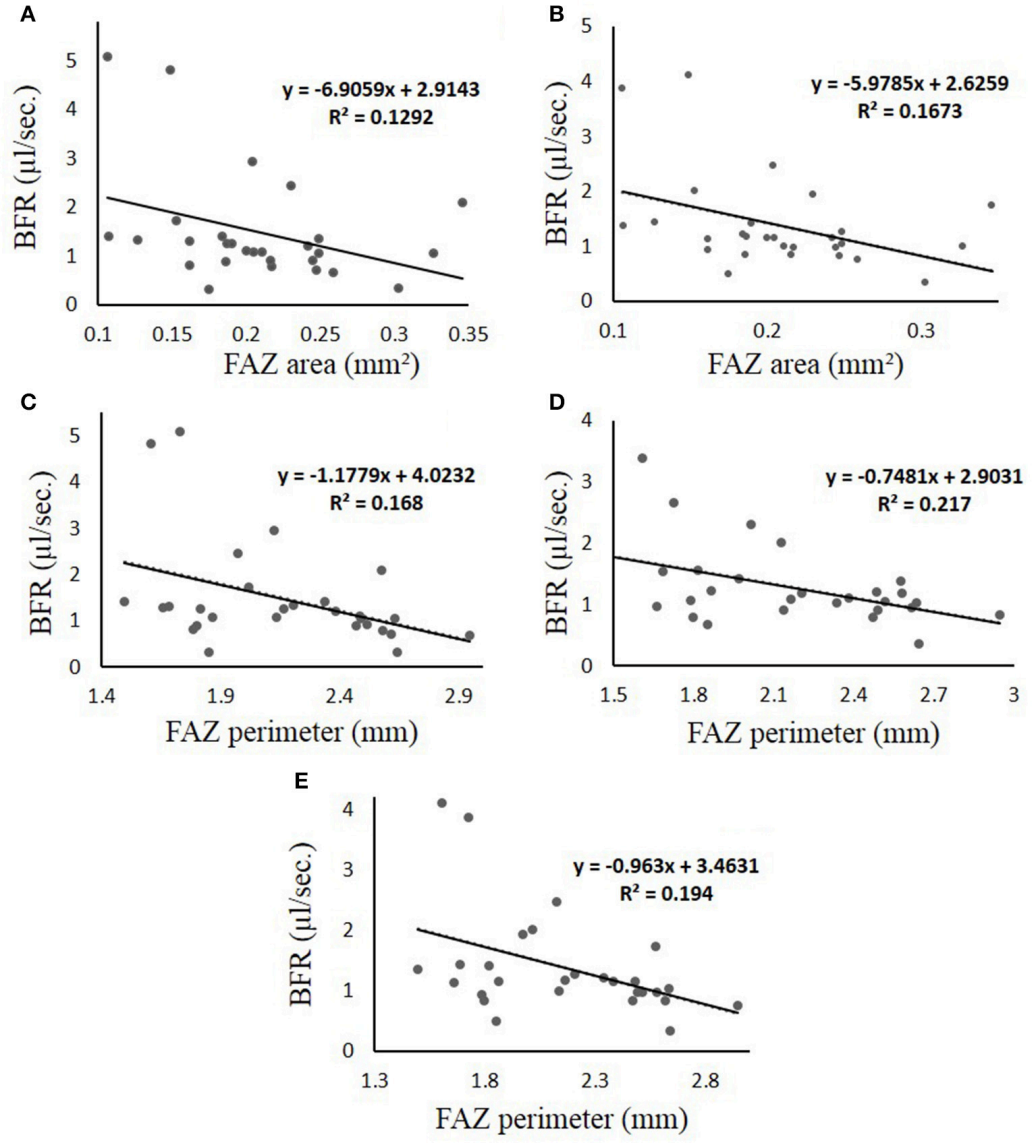
**(A–E)** Linear regression results obtained for the DM group of patients, which is the only group that displayed significance when comparing the BFR to the FAZ characteristics. Each figure is labeled with measured parameters. The line represents thel ine of best fit, of which the equation is displayed in the graph region. In all cases, the “y-axis” represents BFR in μl/s and the “x-axis” represents FAZ area in mm^2^. **(A)** BFR vs. FAZ area in veins **(B)** BFR vs. FAZ area in all vessels **(C–E)** BFR vs. FAZ Perimeter for arteries, veins, and all vessels, respectively. The *R*^2^ is the coefficient of determination, which compares the ratio of the average difference of the data point and the line of the best fit with that of the data point and the average “y” value.

In comparing the BFR with the FAZ characteristics, only the DM group showed a statistically significant linear relationship. These relationships are shown in Figure 5, where the *R*^2^ value and the equation of the line of best fit is displayed on their respective graphs.

## Discussion

In this study, we investigated the FD of the foveal vessel arborization in relation to the morphology of the FAZ and the macular circulation in patients with T2DM. Our results demonstrated the highest FD in the healthy group compared to the DM and MDR groups, with a significant difference between the three groups. Our results are similar to the outcomes presented by Grauslund et al., where the FD of 94 patients with T1DM without proliferative retinopathy were compared with 79 T1DM patients with proliferative retinopathy and found that the group with the most severe disease had lower FD (Grauslund et al., 2010). Similarly, Aliahmad et al. found that the healthy group had higher FD compared with the diabetic group and suggested that a low FD could be a result of the retina being less effective, which may lead to an increased risk of complications like proliferative retinopathy (Mainster, 1990; Aliahmad et al., 2014).

Numerous studies have assessed the vascular complexity (FD) in patients with different types of DM and stages of DR with contradicting findings. A 16-years study that monitored 180 patients with T1DM found that FD of the retinal vasculature generally decreased in these population. It was also found that lower FD could predict neuropathy (Broe et al., 2014). On the other hand, Cheung et al. and Yau et al. found that an increase in vascular FD was associated with an increased incidence of retinopathy (Cheung et al., 2009; Yau et al., 2010). However, all these studies had different study designs and used fundus machines with different resolutions and illumination settings. The quality of the images acquired from the study subjects was also dissimilar. Also, the region of interest for the FD calculation was not the same for all studies. For example, our study analyzed the FD in a 20° FOV while the other studies looked at the vessels at a larger scale. This difference in methodologies may cause contradicting findings among studies. Huang et al. found that the FD must be calculated under very rigorous settings after investigating the reliability of the vascular FD calculated from retinal images acquired with 5 different fundus cameras (Huang et al., 2016). Therefore, when comparing different studies, it is of great importance to consider the study design, the settings used for the analysis of the acquired images and image resolution among other key factors.

The results of our study showed that there were significant differences in FAZ parameters that describe different morphology characteristics between all 3 groups. Results of the FAZ characterization showed no significant differences in the DM group in comparison to the healthy group, while the MDR group had significantly higher FAZ area and perimeter in comparison to the healthy and DM groups. Also, a significant difference in roundness was only observed between DM and MDR groups. These findings might indicate that the FAZ roundness might be a good indicator of DR onset and progression. Intriguingly, as reported in our previous study, the fact that the FAZ area was larger in the MDR group but more asymmetric in the DM group suggest a possible anisotropy in the mechanical properties of the diabetic retina with no retinopathy. This anisotropy may trigger the FAZ elongation in a preferred direction as probably a result of autoregulation (Bates et al., 2018).

The findings in the current study support the loss of symmetry in the FAZ expansion with worsening of the retinopathy condition. Our results are in accordance with the fact that the enlargement of the FAZ area is an indicator of DR onset and progression besides being an indicator of visual prognosis in patients with DME (Bates et al., 2018). It is well known that larger FAZ area is associated with progression of DR, compared to healthy individuals, as reported after using FA (Sakata et al., 2006). Additionally, Optical Coherence Tomography-Angiography (OCTA) studies have found a negative correlation between FAZ area and visual acuity. However, FAZ size is not fixed among individuals; therefore, its normal variation makes assessment of retinal pathology in terms of FAZ size very difficult (de Carlo et al., 2015b; Freiberg et al., 2015; Mammo et al., 2015; Spaide et al., 2015; Takase et al., 2015; Al-Sheikh et al., 2016; Bhanushali et al., 2016; Samara et al., 2016; Tan et al., 2016).

There are many discrepancies in studies targeting retinal blood flow, mainly due to differences in the device used and in the study design. Particularly, the variability in these studies is affected by factors including the recruitment of patients with T1DM diabetes vs. T2DM, controlled vs. poorly controlled diabetes, age, presence or absence of other co-morbidities such as systemic hypertension (Kohner, 1975; Grunwald et al., 1993, 1996; Schmetterer and Wolzt, 1999; Pournaras et al., 2008). Our study population consisted of age-matched groups composed by healthy controls and T2DM subjects with and with no mild DR. The diabetes disease condition in T2DM patients was in control but most of the T2DM patients were hypertensive (under control). The fastest BFR for overall vessels (that is, arteries and veins taken together) was found in healthy subjects, compared with the DM and MDR groups, whereas the slowest BFR was observed in the MDR group. This is in correlation with previous investigations where reduced retinal blood flow and vessel stiffness seemed to be associated with nonperfusion in the vasculature network (Kohner, 1975; Grunwald et al., 1996; Schmetterer and Wolzt, 1999; Sakata et al., 2006; Pournaras et al., 2008).

In comparing the correlation of the BFR and FD for the three groups, a significant correlation between FD and BFR was obtained for the healthy group in all three groupings of the vessels (overall arteries, overall veins and overall vessels). Nevertheless, we did not find any significant correlation for the DM and MDR groups. Also, the BFR and vascular FD among healthy subjects and diabetic patients with and with no DR have not been compared in previous studies. According to our findings, it seems that the flow distribution of the retinal vessels loses complexity while fractal branching does not adequately describe the vascular structures involved in this process.

Although the BFR was lower in the MDR group than in the DM and healthy groups, intriguingly, the correlation between BFR and FAZ was statistically significant with low *R*-squared values only for the DM group. It is possible that our data contain an intrinsically higher amount of unexplainable variability. Also, there was a negative correlation between FAZ perimeter and BFR for the DM group when compared in overall arteries, overall veins and overall vessels. As well as there was a negative correlation between FAZ area and BFR for overall veins and overall vessels. Interestingly, we saw no statistically significant correlation between BFR and FAZ roundness in the DM group. Also, there was no statistically significant correlation between BFR and FAZ in the MDR and healthy groups.

The reduction in BFR in patients with DM may be due to morphological changes of the vascular bed in combination with lack of capability of vascular autoregulation and the decrease of blood fluidity (Ashton, 1974; Sinclair et al., 1982; McMillan, 1989). Our results could be explained by the fact that an increase in FAZ area and perimeter can result in decreased BFV, and consequently a decreased BFR, as demonstrated by the negative correlation observed between BFR and FAZ size. However, our study does not have the full power to prove this outcome as there are many factors that have an influence on microcirculation in diabetic patients with DR, such as duration of disease, changes in ocular biomechanics, and the presence of other concomitant systemic diseases (e.g., controlled hypertension in our diabetic groups) that can contribute to a slower BFR in patients with DR. A statistically significant negative correlation between capillary BFV and FAZ size in T2DM was found in a previous study that used FA as the measurement method (Sakata et al., 2006). However, a correlation between FAZ parameters and BFR in patients with DR or a healthy control group seems to be unavailable in the current literature. Our fractal dimension results also revealed the potential use of this method to quantify the progressive change in DR between the increased and decreased vessel complexity stages. The complexity index of the retinal vascular pattern characterized by fractal analysis may uncover potential regulation of specific markers of disease status.

There are important limitations to the present study that need to be addressed. There was a different sample size for each one of the 3 groups due to the exclusion of eyes because of the poor image quality obtained with the RFI unit in some eyes with media opacities. Also, only subjects with T2DM were included. Consequently, the degree to which our outcomes can be generalized to individuals with Type 1 diabetes is uncertain. Especially, retinal structure and function may be affected by factors such as hyperlipidemia, older age, and hypertension in Type 2 diabetes. Furthermore, we conducted our study in a relatively small sample of patients. A better understanding of the correlation between BFR, FD, and FAZ measurements in a bigger number of subjects and longitudinal studies is devised as a future study.

## Conclusion

There are many structural and hemodynamic parameters that can play a role in the development of DR. Particularly, the development of advanced imaging will facilitate that these quantitative measurements can help with the identification of changes in the retinal structure affected by various diseases with greater precision and detail (Tian et al., 2016). Our results suggest that the FD of the foveal vessel arborization in conjunction with other functional and structural parameters could provide useful information to identify early morphological changes in the retinal tissue of patients with T2DM. The data also lead us to believe that the enlargement and asymmetry of the FAZ area might be related to a lower BFR associated with the onset and progression of DR (Krawitz et al., 2017). Notably, due to the lack of FAZ symmetry observed in DM and MDR eyes, it appears that the distribution of flow within the retinal vessels loses complexity as the vascular structures distributing the flow are not well described by fractal branching. In addition, despite the availability of many different studies about FAZ and DM or FD and FAZ, to our knowledge, there are no known studies that correlate FD with hemodynamic and structural parameters. However, further longitudinal research is warranted to determine how our approach may be used to aid diagnosis of retinal neurodegeneration and vascular impairment at the early stage of DR. There is no doubt that there is a need for a better understanding of structural and hemodynamic parameters which are integrally interdependent. Specifically, the multimodal measurements in our future work would not only provide details of retinal pathophysiology but could possibly contribute as a biomarker in disease staging.

## Author contributions

DC conceived and designed the study. DC, JT, GS, WS, W-HL, AK, NG, SP, and MK performed the study; NB, NM, JT, GS, WF, JS, DC, and MK analyzed the data. JT, DC, WF, JS, SP, and NM: contributed reagents, materials, and analysis tools. MK, NB, NM, JT, DC, WS, and GS contributed to the writing of the manuscript.

### Conflict of interest statement

The authors declare that the research was conducted in the absence of any commercial or financial relationships that could be construed as a potential conflict of interest.
